# LDIAED: A lightweight deep learning algorithm implementable on automated external defibrillators

**DOI:** 10.1371/journal.pone.0264405

**Published:** 2022-02-25

**Authors:** Fahimeh Nasimi, Mohammadreza Yazdchi

**Affiliations:** Department of Biomedical Engineering, Faculty of Engineering, University of Isfahan, Isfahan, Iran; Valahia University of Targoviste: Universitatea Valahia din Targoviste, ROMANIA

## Abstract

Differentiating between shockable and non-shockable Electrocardiogram (ECG) signals would increase the success of resuscitation by the Automated External Defibrillators (AED). In this study, a Deep Neural Network (DNN) algorithm is used to distinguish 1.4-second segment shockable signals from non-shockable signals promptly. The proposed technique is frequency-independent and is trained with signals from diverse patients extracted from MIT-BIH, MIT-BIH Malignant Ventricular Ectopy Database (VFDB), and a database for ventricular tachyarrhythmia signals from Creighton University (CUDB) resulting, in an accuracy of 99.1%. Finally, the raspberry pi minicomputer is used to load the optimized version of the model on it. Testing the implemented model on the processor by unseen ECG signals resulted in an average latency of 0.845 seconds meeting the IEC 60601-2-4 requirements. According to the evaluated results, the proposed technique could be used by AED’s.

## Introduction

Cardiac Arrest (CA), which refers to the abrupt and cessation of adequate circulation, can affect anyone at any time. 30000 CA’s occur outside of hospitals across the UK each year, and currently, fewer than 10% of these cases survive; however, with speedy emergency treatment, chances of survival would increase by 80% [1]. Cardiopulmonary resuscitation (CPR) and Automated External Defibrillators (AED) shocks given within the first four minutes are crucial for survival [2, 3]. Defibrillation is a common treatment for life-threatening ventricular tachyarrhythmia such as Ventricular Fibrillation(VFib) and Ventricular Tachycardia (VT) [4, 5]. VFib and VT rhythms both represent disorganized electrical conduction which originates in the ventricles [6]. Defibrillation delivers a medicinal dose of electrical current to the heart with a device called a defibrillator. This electrical current depolarizes an acute mass of the heart muscle, ends the arrhythmia and allows normal sinus rhythm to be re-established by the body’s natural pacemaker in the sinoatrial node of the heart. Accurate detection of VFib and VT, known as a shockable signal, is crucial in using defibrillators.

Recently, various automated shockable signal detection algorithms based on machine learning (ML) techniques have been proposed. These algorithms consist of preprocessing ECG signals, extracting the features (attributes) from the preprocessed signals, selecting the subset of essential features and feeding them to the classifiers. The main advantage and disadvantages of ML techniques is low complexity and high time consumption and low accuracy, respectively. Deep learning techniques have a lot of benefits for arrhythmia detection. One of the main benefits of these techniques is that they perform the feature extraction automatically, resulting in the increasing of accuracy compared to ML techniques. However, state-of-the-art techniques need to be improved to meet the IEC 60601-2-4 requirements [7].

As mentioned earlier, timely and accurate detection of shockable signals is crucial, so it is advantageous to design and implement systems that help laypersons in applying the proper treatment at the legal time. Hence, the goal of this work is to use a deep learning model to make a shockable versus other non-shockable signal classifications and implementing the optimized model on the desirable processor of an AED.

The LDIAED method was applied to several ECG arrhythmias to assess the capability of this technique to detect shockable signals. The proposed LDIAED algorithm implemented in an AED analyzes the heart rhythm and distinguishes VT/VFib from other rhythms without the need for extensive preprocessing or feature extraction of raw electrocardiogram signals with higher than 99% accuracy and in less than 0.85 ms.

The main contribution of this study is the proposal of a lightweight CNN technique to detect shockable signals sampled with any frequency without the need for pre-processing and implementing the optimized model on the dedicated processor.

In the rest of this paper, after a brief review of related work in the literature review section, a description of VT/VFib signals is given in morphology of shockable signals section. After that, some challenging issues related to the implementation step are provided. The methodology section describes the proposed scheme, and simulation and results section presents an evaluation of the proposed work and compares it with several state-of-the-art techniques. In the end, the implementation of the model on the processor is presented and results were acquired.

## Literature review

For accurate diagnosis of shockable signals, it is highly desirable to have optimized and accurate automated arrhythmia detection algorithms. In the following text, we discuss a few most promising existing automated algorithms designed to differentiate between shockable signals and other signals [8]. Automated algorithms fall into two categories, non-AI techniques and AI-based techniques. In [9], to develop the detection system, many electrocardiogram signals have been analyzed by using Gabor wavelet transform (GWT). Detection performances for all combinations of spectrum feature parameters are evaluated and valuable spectrum features for ECGs are extracted. In AI-based techniques, ML algorithms are used to detect shockable signals. In 2016 Figuera et al. computed a set of 30 VF-detection features related to temporal, spectral, time-frequency and complexity features. These features are then fed to state-of-the-art ML algorithms with built-in feature selection capabilities to determine the optimal feature subsets and finally detection of shockable rhythms [10]. Nguyen et al. extended a set of two features such as Count2 and VF-filter Leakage measure (Lk) to use support vector machine (SVM) model. Then, they supplemented five more features based on a binary genetic algorithm [11]. Sharma et al. used the fuzzy, Renyi and sample entropies from various wavelet coefficients and fed them to SVM classifier for automated classification [8]. Authors in [12] used Digital Taylor-Fourier transform (DTFT) to decompose the ECG signal into various oscillatory modes. The magnitude and phase difference (PD) features are evaluated from the mode Taylor-Fourier coefficients of ECG signal and finally least square support vector machine (LS-SVM) classifier with linear and radial basis function (RBF) kernels are employed for the detection and classification of VT versus VFib, non-shockable versus shockable and VFib versus non-VFib arrhythmia episodes. The proposed algorithm in [13] consists of K-nearest neighbor classifier and an optimal set of 36 features, which are extracted from original ECG using modified variational mode decomposition technique. In [14], a fixed frequency range empirical wavelet transform (FFREWT) filter-bank is introduced for the multiscale analysis of ECG signals. The modes which were evaluated using FFREWT of ECG signals are used as input to a deep convolutional neural network for the detection of shockable ventricular cardiac rhythms. In [15], they proposed a method based on ensemble empirical mode decomposition to decompose the ECG signal and classified with decision tree classifier and SVM for discriminating the VT/VFib conditions using informative ranked features. In [16], the signal is decomposed with the wavelet transform, empirical mode decomposition and variable mode decomposition approaches and twenty-four features are extracted to form a hybrid model from a window of 5 second length. Acharya et al. have proposed an 11-layer convolutional neural network model for automated differentiation of shockable and non-shockable ventricular rhythms [17]. The study in [18] was to assess the feasibility of feeding two-dimensional (2D) time-frequency maps of electrocardiogram (ECG) segment into deep convolutional neural network to automatically detect shockable signals with emphasis on optimizing the convolutional neural network model and shortening the analysis segment. The objective of [19] was to apply a deep-learning algorithm using convolutional layers, residual networks, and bidirectional long short-term memory to classify shockable versus non-shockable signals in the presence and absence of CPR artifact components associated with the mechanical activity of compressions and ventilation of the heart.

The final goal of the mentioned techniques is implementing the algorithm on AEDs, so recently, researchers are trying to design lightweight algorithms or optimize the existing classification techniques. Authors in [20] proposed a real-time arrhythmia discrimination algorithm using time domain analysis technique and ported it to FPGA and fabricated the AED prototype. Moura et al. in a complete study, managed to develop a mobile application to assist the diagnosis of different arrhythmias and quantized and implemented their proposed CNN classification algorithm [21]. Sparkfun Edge Apollo 3 (a low-power microcontroller board designed specifically for long battery life) used as the portable hardware for the implementation of the classification technique designed by [22]. Authors in [23]employed a novel Knowledge Distillation (KD) method to uniquely compress a baseline DNN model to achieve significant compress gain and also pruned and quantized the compressed model to implement it on wearable ECG devices. In more related work, authors in [24] used identified peaks and heart rates as input features to two hierarchical SVM classifiers to separate VFib, VT and normal signals. Finally, the Raspberry pi board is used as a hardware platform to embed the proposed algorithm into an AED system.

## Morphology of shockable signals

**Fig 1:**
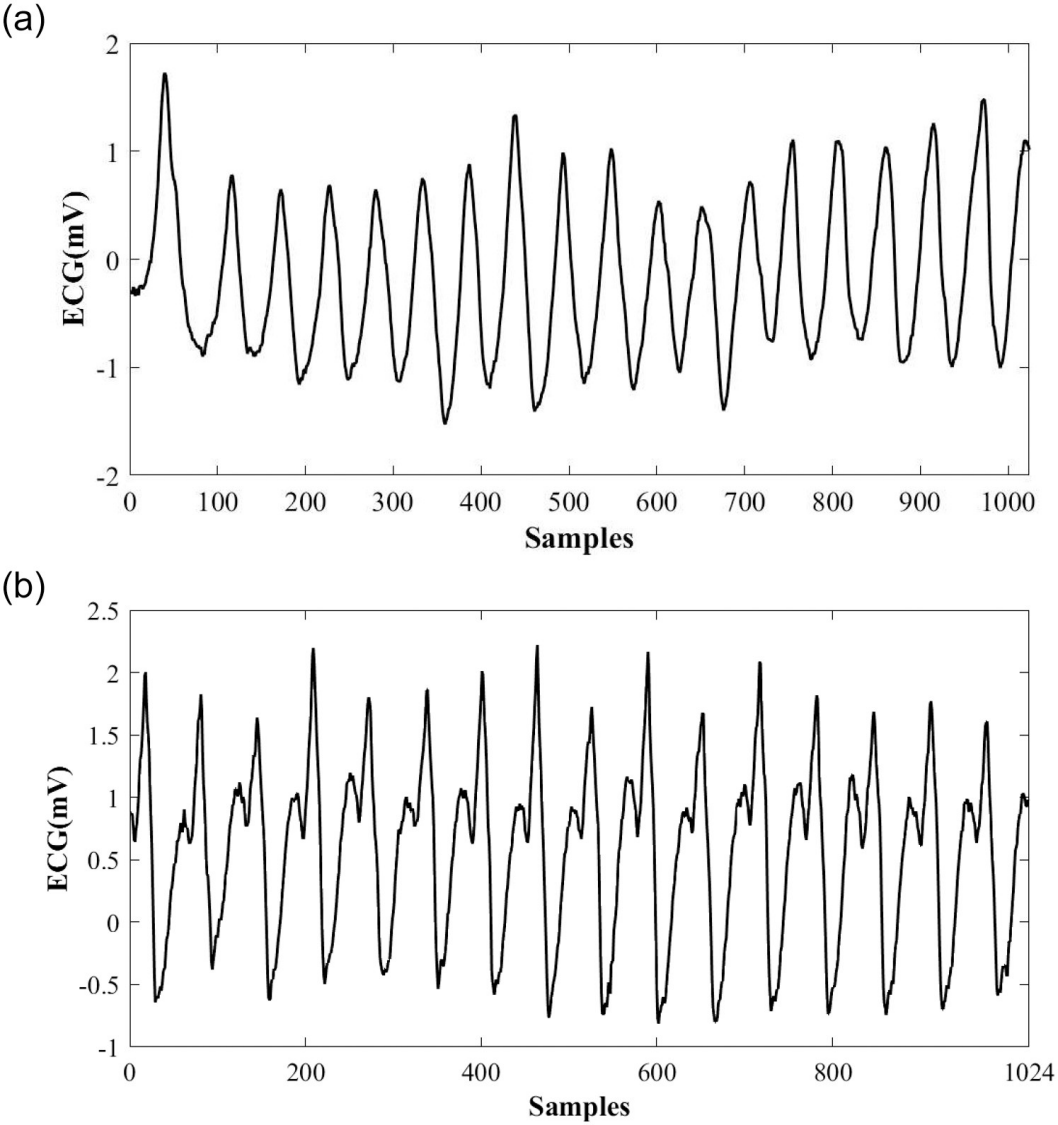
Shockable ECG signals. A: An example of a VFib ECG signal (top). B: An example of a VT ECG signal (bottom).

A healthy heart is usually controlled by electrical signals which start in the sinoatrial node often called heart’s natural pacemaker; then it moves down to the atrioventricular node. This signal makes the ventricles contract and move the blood along. When the electrical signals in the ventricles move the wrong way; the situation is called VT. The disordered heartbeats stop the heart chambers from properly filling with blood. In some cases, this situation can lead to Vfib, which causes very rapid and uneven heartbeats. Vfib and VT are life-threatening cardiac signals that result in inefficient ventricular contractions. A pulseless VT is when a ventricular contraction is so quick that the heart could not be refilled, resulting in an unnoticeable pulse. In both cases, body tissues do not receive sufficient blood flow. Although VFib and VT have different pathological phenomena and ECG morphology, the Advanced Cardiac Life Support (ACLS) managements of both are essentially the same. ACLS responses to VFib and pulseless VT within a hospital will probably be performed using a cardiac monitor and a manual defibrillator. Thus, the ACLS provider must read and analyze the rhythm. Due to human faults, it is better to use automated defibrillators. As it can be seen in Fig 1A, rules for VFib include a bizarre shape of the QRS complex (disorganized electrical activity), a rapid heart rate, no P waves and no PR interval [25]. Rules for VT usually include regular R-R intervals(not always), an undetermined atrial rate; ventricular rate between 150 and 250 beats per minute and QRS complexes are not preceded by P waves as in Fig 1B. The PR interval is not measured since this is a ventricular rhythm, and the QRS complex lasts for more than 0.12 seconds. The QRS will usually be wide and bizarre and it is typically challenging to see a separation between the QRS complex and the T wave [26].

## Challenging issues in model implementation

Treating out-of-hospital cardiac arrests is highly challenging due to their unpredictability, the urgency of intervention and high sensitivity requirement in the detection of life-threatening arrhythmias [27]. Furthermore, if defibrillation is performed during the first minute of collapse, the survival rate is as high as 90% [28]. When defibrillation is postponed, survival rates decrease to approximately 50% at five minutes, approximately 30% at seven minutes, approximately 10% at 9 to 11 minutes, and approximately 2% to 5% beyond 12 minutes [29].

According to the mentioned reports, the implemented model on the AED processor only has less than one minute time to correctly detect the shockable signal to reach the survival rate of 90%. Moreover, the sensitivity of the implemented model should be above 90% according to IEC 60601-2-4 requirements. In consequence, the main challenges in AEDs are the detection time and the accuracy of the shockable signal and specificity of non-shockable signal detection.

## Methodology

**Fig 2:**
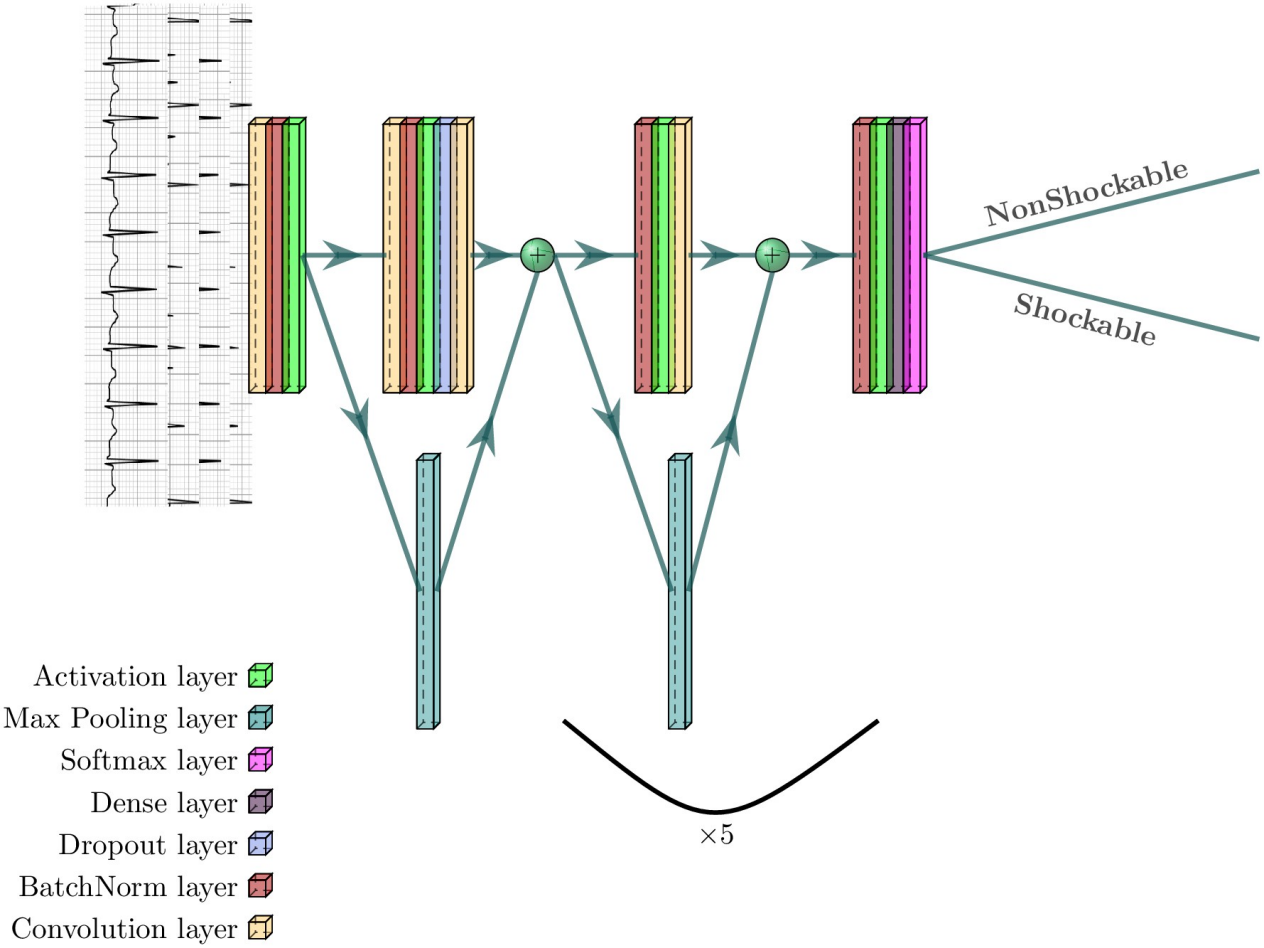
Deep Neural Network architecture.

Different techniques are being used in AED’s. Machine learning and deep learning techniques are the most excellent state-of-the-art techniques that are active in those devices. DNNs have proven that they can recognize patterns and learn useful features from ECG signals without the need for preprocessing or feature extraction techniques. The methodology used in this work is based on a lightweight deep learning algorithm. In our work, to train and evaluate the model, we used three databases and categorized those ECG signals into two classes, shockable and non-shockable (normal, paced, atrial fibrillation and etc.). As shown in Fig 2, our approach is novel in using a 9-layer network in an end-to-end manner simultaneously discriminating between shockable signals and non-shockable ones, all of which are enabled by our dataset. In the proposed technique, to achieve a high classification performance no preprocessing of ECG data such as Fourier or wavelet transforms is done.

We extracted numerous 1024 sample ECG’s containing shockable and non-shockable signals to construct the training and test dataset. We used a convolutional DNN to differentiate between shockable and non-shockable signals, which take the raw ECG data as input and puts one prediction out every 256 samples. In various automated systems, different segment sizes (10s,8s,6s,5s and 2s) are used for shock or non-shock advice. The short segment size is always desirable for fast inference. In this work, we used the segment size of 256 samples equal to 0.9s or 1.4s (250 Hz or 360 Hz). It is important to note that although we used a short size, our classification results are better than previous works. To find the best configuration of hyperparameters which will give us the best accuracy we used the grid search technique. There are two kind of hyperparameters in DNNs which need to be tuned before the training phase, hyperparameters related to network structure (dropout rate, network weight initialization and activation function) and hyperparameters related to training algorithm like, learning rate, momentum, batch size and number of epochs [30]. In our work, we found the best number of hidden layers and neurons manually. In this technique the starting point of the search was the work done in [31]. We continued reducing the number of layers until the accuracy began to decrease. We utilized shortcut connections similar to the residual network architecture to make the optimization of such a network manageable. The network consists of 6 residual blocks with the maximum of two convolutional layers per block. The convolutional layers in this network extract features with 32 filters of a width of 16, and the stride of filters alternate between four and one. Grid search technique for finding the best choice for batch size and number of epochs resulted in 32 and 100 respectively. This technique suggested Adam optimization algorithm to update network weights iteratively in the training phase. The learning rate, one of the most important hyperparameter was tuned to 0.001 and was reduced by a factor of 10 when the set loss stopped improving for two successive epochs. For adapting the network weight initialization, all of the available techniques where evaluated by grid search technique and “he_normal” was chosen. To adopt the pre-activation block design, normalization and a rectified linear activation layer are used before each convolutional layer. To tune the dropout hyperparameter a range between 0.0 and 0.9 was selected for the grid search algorithm and after searching, 0.2 was chosen to avoid overfitting. After building and tuning the hyperparameters of the model, the training subset of the datasets is used to train the model and at the end, the model with the lowest error (highest accuracy) is used for evaluation.

To estimate the skill of the proposed model on unseen data, 10-fold cross validation approach is used. This approach divides the set of 16062 segments into 10 groups of approximately equal size. The first fold is treated as a test set and the method is fit on the remaining nine folds; finally the accuracy is averaged over all test groups [32].

## Simulation and results

### Dataset

The data utilized in this work were obtained from three sources; namely, MIT-BIH database [33], MIT-BIH Malignant Ventricular Ectopy Database(VFDB) [34] and a database for ventricular tachyarrhythmia signals from Creighton University (CUDB) [35, 36]. The information regarding the databases used in this study is presented in the table below.

### Performance evaluation metrics

As one could see in Table 1 the classes are imbalanced, so to combat this problem, different metrics in addition to accuracy have been used to give a more accurate result [37]. To compare the LDIAED technique with the state-of-the-art techniques, we calculated the Accuracy (Acc) (Rate of correct classifications), Sensitivity (Se) (ability to correctly identify shockable rhythms), Specificity (Sp) (ability to recognize non-shockable rhythms) and F_1 score (the measure of the model’s accuracy) from the confusion matrix for test records.

$$\text{Acc}\;(\%)≜\frac{TP+TN}{TN+TP+FP+FN}\times 100\%.$$

$$\text{Se}\;(\%)≜\frac{TP}{TP+FN}\times 100\%.$$

$$\text{Sp}\;(\%)≜\frac{TN}{TN+FP}\times 100\%.$$

$$\text{F\_1}≜\frac{2TP}{2TP+FP+FN}$$

**Table 1:** Used databases.

DATABASE	frequency(Hz)	NUM of shockable rhythms	NUM of other non-shockable rhythms
*MIT* − *BIH*	360	0	6587
*VFDB*	250	1239	4192
*CUDB*	250	669	3375

Above, a True Positive (TP) is an outcome in which the model correctly predicts the shockable signals, True Negative (TN) is an outcome in which the model correctly predicts other non-shockable signals. A False Positive (FP) is an organized or asystole that has been incorrectly classified as a shockable rhythm and a False Negative (FN) is a VF or VT associated with the cardiac arrest that has been incorrectly classified as non-shockable signal.

### Experimental results and discussion

Timely detection of shockable signals is crucial; hence, it is of utmost importance to capture the shockable signal within a short duration of ECG signal [14]. In this study, a novel CNN-based algorithm for the automated detection of shockable and non-shockable ECG episodes is presented. We managed to reduce the complexity (number of layers) of the algorithm by 73.5% compared to the state-of-the-art model used in [31] and keep the sensitivity and specificity of the detection algorithm above the boundary needed by IEC 60601-2-4 requirements [7]. The LDIAED technique can detect shockable signals with a sensitivity of 96.13% and can detect other non-shockable signals with a specificity of 99.64%. The fraction of good predictions, which refers to the accuracy of the model, is 99.1%. As shown in the confusion matrix demonstrated in Table 2, from 1606(test partition) segmented ECG’s collected from the mentioned databases, only 12 segments are misclassified. From these 12 segments, seven segments belong to the shockable class, misclassified as non-shockable signals. As mentioned in [38], not advising shock for a patient with shockable signal might lead to their death, but fortunately, in this study, the rate of this kind of misclassification is only 3.87%. A quick review of this kind of misclassification unveiled that this misclassification entirely appears to be very sensible. In many cases, lack of anatomical considerations, movement artifacts, limited signal length, or having a single lead limited the derivation that could be concluded from the data, making it challenging to certainly reveal whether the annotating cardiologists and/or the algorithm was correct.

**Table 2:** Confusion matrix.

		True Class	
		shock	nonshock
Predicted Class	shock	*TP* = 174	*FP* = 5
	nonshock	*FN* = 7	*TN* = 1420

LDIAED method segmented the 1024-sample ECG into four 256-sample segments to capture the shockable signals. Algorithm performance is evaluated by its sensitivity and specificity and is compared with other techniques collected in Table 3. It becomes clear that the LDIAED method outperforms the state-of-the-art techniques without the need for preprocessing or feature engineering and is above the IEC requirements. The main point of this study is that the LDIAED CNN model can advise shock or non-shock for a short ECG signal in two seconds by using a lightweight CNN. The other novelty of our work is in the using of three diverse databases with different frequencies consisting of a total of 16062 ECG segments (1908 shockable and 14154 non-shockable) to train and test the model.

**Table 3:** State-of-the-art deep learning methods used for automated detection of shockable ECG signals.

Author/Year	Performance	Used Databases
Okai et al. [9]/2020	*AUC* = 0.967	AHADB, MITDB, CUDB
Nuguyen et al. [11]/2018	*Acc* = 95.9%, *Sp* = 96.8%, *PPV* = 87.6%	MITDB, CUDB
Sharma et al. [8]/2020	*Acc* = 98.9%, *Se* = 99.08%, *Sp* = 97.11%, *AUC* = 0.99, *F*_1 = 0.994	MITDB,CUDB
Tripathy et al. [12]/2018	*Acc* = 89.81%, *Se* = 86.38%, *Sp* = 93.97%	CUDB,VFDB
Hai et al. [13]/2021	*Acc* = 99.2%, *Se* = 96.7%, *Sp* = 99.7%	CUDB,VFDB
Panda et al. [14]/2020	*Acc* = 99.03%	CUDB,VFDB
Mohanty et al. [15]/2021	*Se* = 97.94%, *SP* = 99%, *Acc* = 98.69%	CUDB,VFDB
Sabut et al. [16]/2021	*Acc* = 99.2%, *Se* = 98.8%, *Sp* = 99.3%	CUDB,VFDB
Acharya et al. [17]/2018	*Acc* = 93.2%, *Se* = 95.32%, *Sp* = 91.04%	MITDB,CUDB,VFDB
Lai et al. [18]/2020	*Acc* = 98.82%, *Se* = 95.05%*Sp* = 99.43%	MITDB,CUDB,VFDB,AHADB
Hajeb et al. [19]/2021	*Se* = 95.21%, *Sp* = 86.03%	CUDB,VFDB,SDDB
**Proposed**	**Acc = 99.1%**, *Se* = 96.13%, *Sp* = 99.64%, *F*_1 = 0.996	MITDB,CUDB,VFDB
**IEC 60601-2-4 requirements [7]**	*Se* > 90%, *Sp* > 95%	—–

## Hardware implementation of the proposed algorithm

To use the proposed model in practice, this model must be loaded in a dedicated processor to be used in an AED. Raspberry Pi, the intended processor, is a low-cost, tiny desktop computer that is ideal for programming. With its controllable input-output pins, sensors and other hardware can be read out and controlled very easily. The raspberry pi has ARMv6 700 MHz single-core processor, a VideoCore IV GPU, and 512MB of RAM and it uses an SD card for its operating system and data storage.

To run the proposed model on raspberry pi, a set of tools called tensorflow lite is used. These tools support diverse language, have high performance, support multiple platforms and are optimized for on-device machine learning. With these tools the proposed model is converted to a special efficient portable format identified by the .tflite file extension. This conversion reduces the size of the model and increases the speed of inference that enables tensorflow lite version of the model to execute efficiently on devices with limited compute and memory resources. In the proposed work to reduce the model size more, different optimizations such as quantization are applied before conversion. Quantization reduces the precision of the numbers used to represent a model’s parameters, which by default are 32-bit floating-point numbers. Optimization and conversion reduced the model size and latency with minimal or no loss in accuracy [39]. After loading the tensorflow lite model on the raspberry pi minicomputer, predictions according to the inputs are done.

As shown in Table 4, different quantizations are applied to the model. Post-training float16 quantization converts model weights to 16-bit floating-point values and post-training dynamic range quantization converts model weights to 8-bit precision.

**Table 4:** Effect of different quantization techniques on model inference.

Quantization technique	Accuracy	Latency(ms)	size
Post-training float16 quantization	99.1%	0.832	252 kB
Post-training dynamic range quantization	98.9%	0.845	142 kB
Default(no optimization)	99.1%	1.444	473 kB

Evaluating the proposed model with unseen ECG segments on the server-side results in an accuracy of 99.1%. The model size before any optimization and conversion was 1.551 MB. Optimization and conversion, as seen in the table below, result in smaller model sizes and faster computation, making this model suitable for AED’s. As it is expected, the accuracy of the different optimized versions of the proposed model, are identical to the accuracy of the primary model or have a slight loss, but as a great effort, the size of the model reduces significantly compared to the primary model. So according to the results shown in the table below, the best model suitable for implementing on raspberry pi is the post-training dynamic range quantization plus conversion version of the model, which has the smallest size and the same latency and nearly the same accuracy compared to other versions.

## Conclusion

The applied algorithm presented in this study managed to detect shockable arrhythmias and differentiate them from non-shockable signals in a 1.4-second ECG signal with an automated method. The privilege of the proposed technique is its lightweight end-to-end learning procedure that combines feature extraction with the classifier. The proposed algorithm meets the IEC60601-2-4 requirements. When the device reached a shock or no shock decision, the accuracy was high, with 96.13% sensitivity for shockable rhythms and 99.64% specificity for other non-shockable rhythms.

Another outstanding endeavour of this study is the implementation of the optimized version of the proposed classification method on the raspberry pi minicomputer as a part of an AED. Evaluating the implemented model on the raspberry pi by unseen segments resulted in an average detection time of 0.845 seconds and accuracy of 98.9which meets the IEC60601-2-4 requirements. Considering the lightweight proposed model, real-time feature and the accuracy of the implemented model, it is concluded that the proposed technique outperforms state-of-the-art techniques, and could be used in commercial AEDs.
